# Formation of a unique structure during microsporogenesis in *Tinantia anomala* (Commelinaceae) anthers

**DOI:** 10.1007/s00709-016-0990-y

**Published:** 2016-06-16

**Authors:** Krystyna Winiarczyk, Joanna Gębura

**Affiliations:** Department of Plant Anatomy and Cytology, Maria Curie e Skłodowska University, Akademicka 19, 20033 Lublin, Poland

**Keywords:** Microsporogenesis, Tapetum, Microspore, Pollen grain, Pollen wall, *T. anomala*

## Abstract

The analysis of microsporogenesis in *Tinantia anomala* revealed a unique ring-shaped structure assembling pollen grains into large aggregates. The heterogeneous ring was composed of several segments and was dominated by lipid compounds. Although the ring is a product of the tapetum, such a structure has not been described yet. The authors put forward some hypotheses for elucidation of the role of this structure in the process of male gametophyte formation.

## Introduction

The pollen grain is a male gametophyte and represents the generative phase in the life cycle of angiosperms. The development of pollen in flowering plants consists of three stages: (1) differentiation of sporogenous cells and meiosis (microsporogenesis), (2) post-meiotic development of microspores, and (3) microgametogenesis (Chaudhury et al. 1993; Gómez et al. 2015). Finally, pollen grains can be tricellular or bicellular and serve as a functional male gametophyte. An important role in the development of pollen is played by sporophytic anther tissues, in particular the tapetum, which is indispensable for regulation and coordination of the development processes in the pollen sac. The tapetum is characterised by high metabolic activity; additionally, the process of programmed cell death (PCD) takes place in this structure. A multiple proteolytic system, which is required for the release of materials needed for the development of pollen grains, is involved in the decay of the tissue. The materials are gradually utilised for nourishment of the sporogenic tissue, production of the callose wall, and finally formation of a richly sculptured sporodermal wall (Owen and Makaroff 1995; Quilichini et al. 2015). Immunolocalisation studies have supported a model in which sporopollenin-producing enzymes are engaged in a metabolon within the tapetum (Jiang et al. 2013; Lallemand et al. 2013). The course of physiological degradation of the tissue may take from several days to several weeks, but complete disappearance of the tapetum is noted at the time of pollen release. The tapetum disappears because resorption or degeneration occurs at different stages, but no later than the first haploid mitosis (Liu and Fan 2013; Pacini 2010). In some species, the tapetum serves additional specialised functions, such as production of tryphine, pollenkit or elastoviscin. Elastoviscin occurs in orchids with tetrads (Hesse 1979; Pacini and Hesse 2002). The viscin substance is formed by the cytoplasm of the tapetum. Before anthesis, its droplets fuse and flow into the loculus, gluing the pollen grains together. Pollenkit is a hydrophobic layer composed mainly of lipids and carotenoids derived from plastids and cytoplasmic degeneration (Pacini and Hesse 2002). In many cases, the products of degeneration remain in the loculus and are deposited on the exine surface immediately before pollen dehydration (Ariizumi and Toriyama 2011). The *Tinantia anomala* plant, which is the subject of our research, exhibits an amoeboid tapetum. The cells in this type of tapetum may discontinue at different stages of microspore development, and fusion of their cytoplasm gives rise to the syncytium phase. The content of periplasmodium-forming protoplasts fills the entire loculus, which contains pollen grains, providing a more efficient way of nourishing them. The amoeboid anther tapetum contains hydrophobic material derived from the elaioplast and cytoplasmic lipids (Hesse 2010; Furness and Rudall 1998). The existing knowledge concerning the development of the anther and pollen grains as well as structural changes that accompany these processes is extensive. Additionally, the use of modern methods and research tools provides new genetic and molecular information. Apparently, all the observations at the structural level have already been described. However, the species investigated in this study has provided new information. In this paper, we show the presence of a new structure, which was identified in *T. anomala* with standard microscopic methods. Description of the structure and an attempt at elucidation of its role in the formation of pollen grains are the aim of this study.

## Materials and methods

Anthers of *T. anomala* (Torr.) C.B. Clarke were used in our research to make all microscopic preparations. The plants were grown at 23 °C on a universal, slightly acidic pH 5.5–6.5 soil, under a normal photoperiod depending on the season. The plants were cultivated in a greenhouse in the Botanical Garden of University of Maria Curie-Skłodowska. Herbarium specimens were deposited in the Botanical Garden University MCS catalogue number 1405, in resources from 2005. The observations were carried out on flower buds collected in different developmental stages: the stage of dehydrated microspores embedded in the amoeboid tapetum, the stage of mature pollen grains and microspores with a visible ring structure, the anthesis stage and the anther dehiscence and pollen release stage.

### Preparation of anther samples for LM

Fresh buds of different sizes were collected and then anthers were isolated. In buds which measured 10–12 mm were observed free microspores without the calose wall and in the anthesis stage were observed mature pollen grains. The anthers were stained on a glass slide with 1 % acetocarmine (1 % solution of carmine in 45 % acetic acid) for 10 min. Next, the stained anthers were crushed with a coverslip in a drop of 50 % glycerine. Staining designed to identify the presence of proteins, polysaccharides and lipids were carried out according to standard methods (Gerlach 1977). The crushed anthers were analysed and imaged using the Olympus BX61 microscope equipped with the Olympus Digital Colour camera DP 72 and Olympus CellSens imaging software version 1.5 (Tokyo, Japan).

### Ultrathin sections for transmission electron microscopy (TEM)

Anthers in various developmental stages were fixed in 1 % glutaraldehyde (GA) (1 % (*v*/*v*) GA in 0.1 M phosphate-buffered saline (PBS) pH 6.8 in the presence of 2 % (*w*/*v*) saccharose) for 24 h and washed in a buffer solution. Next, the anthers were post-fixed with 1 % osmium tetroxide (1 % (*v*/*v*) OsO_4_ in the same buffer). After dehydration in an alcohol series, the anthers were transferred to acetone and embedded in acryl resin LR White (Sigma-Aldrich). Ultrathin sections of 60 nm were obtained with an ultramicrotome (Reichert Ultracuts), collected on copper grids and then double-stained with 2 % (*w*/*v*) uranyl acetate and Reynold’s reagent. Observations and image capture were performed with a LEO–Zeiss 912 AB electron microscope (Oberkohen, Germany).

### Scanning electron microscopy (SEM)

The plant material was fixed in 2.5 % GA in 0.2 M sodium phosphate buffer (pH 6.8), washed in distilled water and dehydrated through increasing concentrations of ethanol (Hayat 2002). The dehydrated specimens were then dried in a critical point dryer (Denton Vacuum, Moorestown, NJ, USA) using liquid CO_2_. The fully dried samples were then mounted on aluminium stubs using adhesive carbon tabs and sputter coated with gold (Hummer 6.2 Sputter Coater, Anatech USA, Union City, CA, USA). The samples were analysed under a scanning electron microscope (LEO1430VP) with an accelerating voltage of 15 kV and equipped with a Bruker Quantax 200 X Flash EDX Spectrometer System attached to a Zeiss EVO 50 Variable Pressure SEM at 15 kV, using INCA-Mapping software (Billerica, MA, USA).

### Fluorescence microscopy

Ring autofluoresensis was observed on a laser scanning confocal microscope LSM780 Zeiss with ZEN2010 data acquisition software using a Plant Apochromat 63x/1.40. Fluorescence emission was recorded in the range of 500–560 and 410–460 nm, respectively. Both lasers worked at 2 % power to avoid photobleaching.

## Results

**Fig. 1:**
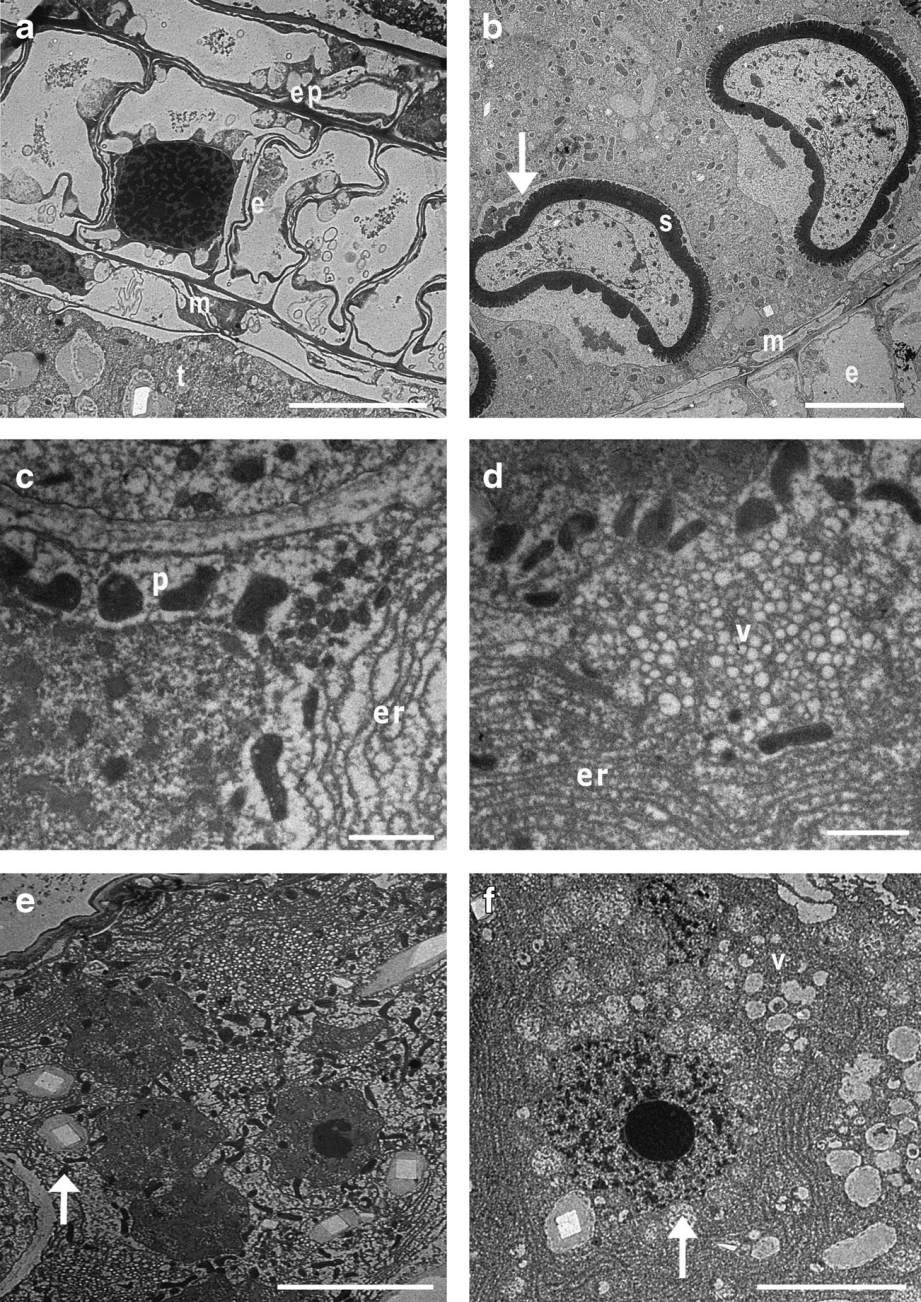
Anatomy of the *T. anomala* anther collected from flower buds with a length of 3 mm (**a**, **b**) 10 mm (**c**, **d**), and 15 mm (**e**, **f**). **a** Fragment of the anther wall of the visible layer of the endothecium (*e*), the middle layer (*m*), and the ameboid tapetum. **b** Fragment of the anther locule with dehydrated uninucleate microspores (*arrow*). Anther locule filled with plasmoidal tapetum with numerous organelles and a few nuclei; around, there are numerous plastids and loosely arranged endoplasmic reticulum threads. **c** Fragment of the tapetum with an abundant network of ER threads and an aggregation of very small vacuoles. **d** Around the tapetal nucleus, there are visible abundant electron-dense structures (*asterisk*) and cross-section of tapetal raphides (*arrow*). **e** Fewer osmophile structures around the nucleus in the syncytium; in the cytoplasm, there are vacuolar vesicles of different sizes. *Bar* = **a**—2 μm; **b**—5 μm; **c**, **d**—1 μm; **e, f**—5 μm

**Fig. 2:**
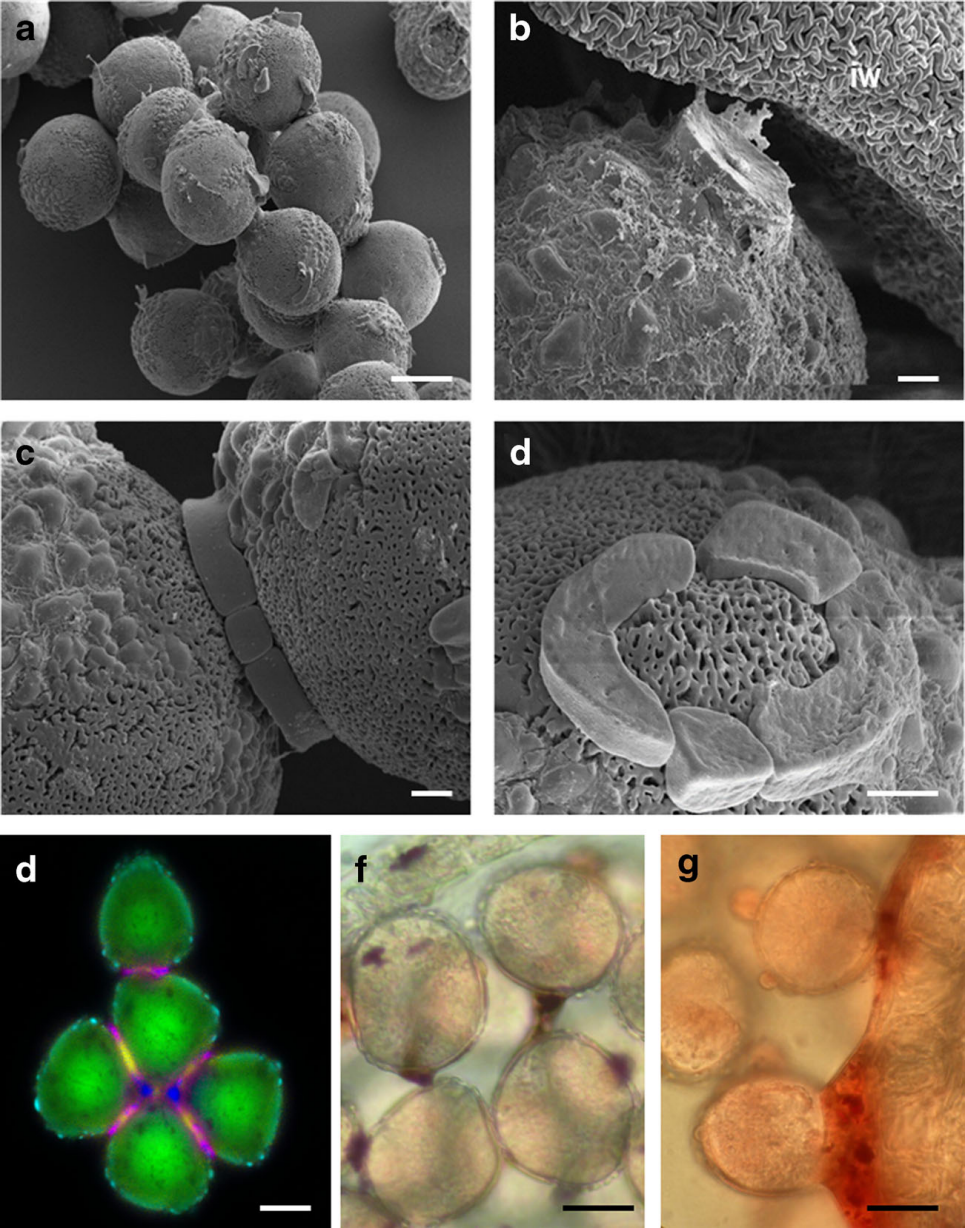
Pollen grains of *T. anomala* observed in 15-mm flower buds. **a** Numerous pollen grains aggregated together by ring structures. **b** Pollen grains connected by a ring with the inner wall of the anther. **c** Two pollen grains connected by a ring. **d** Morphological structure of the ring. **e** Autofluorescence of pollen grains observed in fluorescence microscopy. **f** Pollen grains stained with Sudan III. **g** Sudan IV. *bar* = **a**—10 μm; **b**, **c**, **d**—1 μm; **e**—20 μm; **f**, **g**—10 μm

**Fig. 3:**
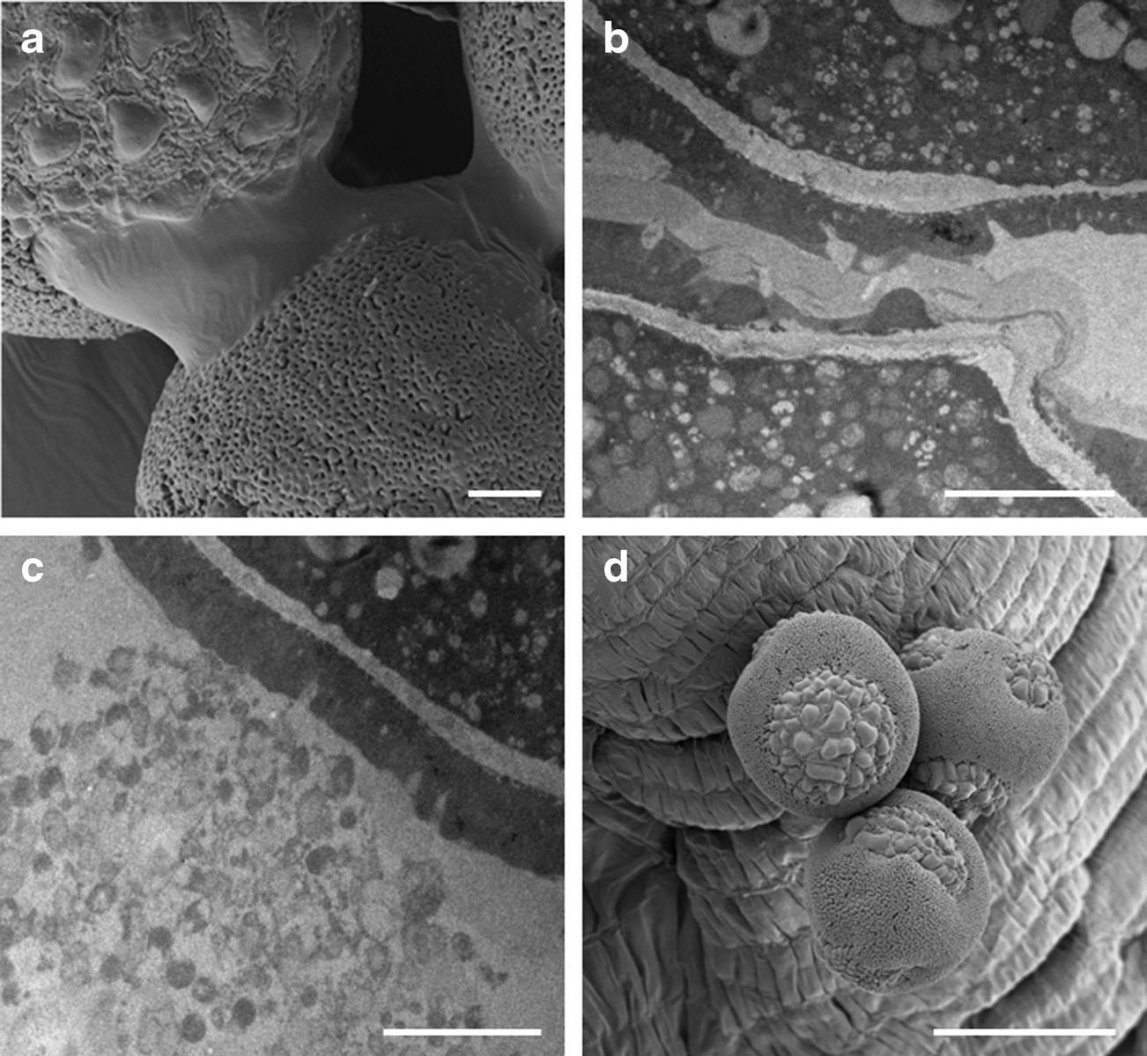
The contents of the anther before dehiscence (**a**, **b**, **c**) and at the anthesis stage (**d**). **a** The change of the state of the ring between pollen grains from the solid to liquid form. **b** The same state observed on a TEM micrograph. **c** Membranes remaining after degeneration of tapetal organelles. **d** Hydrated pollen grains in the anther in the anthesis stage. *Bar* = **a**, **b**, **c**—2 μm; **d**—20 μm

The anatomical observations showed the *T. anomala* anther wall in 3-mm long flower buds was composed of large endothecium cells with large vacuoles and a middle layer (Fig. 1a). The interior of the pollen sacs was filled with an ameboid tapetum with loosely arranged dehydrated uninucleate microspores resembling a “boomerang”. The microspores were embedded in an electron-transparent space separated from the cytoplasm by a single-layer plasma membrane forming a capsule. Within the space, there were irregularly shaped deposits (fibrillar electron-dense material). The periplasmodium cytoplasm contained numerous cell organelles. The microspores were surrounded by a thick osmophilic sporodermal wall with three highly conspicuous, swollen pori. The pollen grain was sulcate with a spinulose or verrucose exine surface; however, there was no visible intine layer at that time. At the sites of the pori, the exine formed large, spherical deposits of osmophilic sporopollenin. In the other parts surrounding the pollen grain, the exine wall was formed of regularly arranged rod-like columellae and a foot layer (Fig. 1b). In 10-mm long flower buds, changes in the ultrastructural organisation of the tapetum were noted. In the ameboid tapetum, large nuclei usually surrounded by osmophilic plastids were observed. Furthermore, clusters of mitochondria, numerous rough endoplasmic reticula (Fig. 1c) and clusters of numerous tiny vacuoles (Fig. 1d) were present in the periplasmodium. The sizes of the tapetal nuclei were varied and their diameter ranged from 4.5 to 6.5 μm. There were also larger nuclei, which had probably undergone polyploidisation (max. diameter 16.5 μm). The pollen sacs in 15-mm long flower buds were filled with an amoeboid tapetum with numerous cell nuclei. They were surrounded by numerous osmophilic, elongated structures. Moreover, the tapetum contained relatively large tapetal raphides (calcium oxalate crystals) (Fig. 1e). In some anther locules, signs of periplasmodium degeneration were evident; there were numerous different-sized vacuolar vesicles and structures with lower osmophilicity surrounded the nuclei (comparison of the structures surrounding the nuclei—Fig. 1c, d, e). The appearance of the nucleus changed as well—there were clusters of electron-dense chromatin and a strongly osmophilic, regularly shaped nucleolus (Fig. 1f). Between the pollen grains, there were ring-shaped structures, which assembled numerous pollen grains into large aggregates (Fig. 2a). Besides the large pollen grain aggregates, single pollen grains were aggregated with the inner anther wall (Fig. 2b). All grains inside the pollen sac were attached to each other by the ring and with the inner anther wall. The rings consisted of a few (4–6) different-sized segments (Fig. 2c, d). The sizes of five rings were measured; the mean length of the segments ranged from 6.5 to 14.8 μm. Observations of the structure under a confocal microscope showed that the ring was not homogeneous and consisted of various components. Autofluorescence of the ring revealed that it comprised domains characterised by a varied capability of visible light excitation (Fig. 2e). The standard cytochemical assays used revealed the presence of sudanophilic compounds (Fig. 2f, g). In turn, assays detecting the presence of polysaccharides and proteins yielded negative results. The ring was highly ephemeral and persisted for a short time (hardly measureable in the investigations). Initially, its state of matter changed from the solid into the liquid form (Fig. 3a) until complete disappearance. The TEM observations indicate that the liquid form of the ring was transparent, i.e. permeable to electrons (Fig. 3b). Anthers with mature grains in the locule contained fragments of degenerated cellular organelles. The ring was completely degraded immediately before anther dehiscence. TEM electronograms showed visible amorphous structures. Between pollen grains, there were membrane fragments with different sizes and a varied transparency degree, membrane clusters and residues of digested cell structures (Fig. 3c). The *T. anomala* pollen was surrounded by a bilayered sporoderm. The outer exine layer was highly osmophilic, and the intine layer contained small cellulose-pectin fibrils. The cytoplasm of the pollen grains had numerous organelles. During anthesis, dehiscing anther walls showed locules filled with mature, hydrated pollen grains with three large, swollen pori (Fig. 3d).

## Discussion

The ring observed in *T. anomala* is an obviously unique structure: it has a regular, segmented structure, it appears at a specified site and time and finally it completely disappears, i.e. it is fully degraded and leaves no recognisable fragments. Amongst the known tapetum products, pollenkit has a lipid character; however, it does not occur in such regular forms. In material fixed chemically for TEM analysis, diffuse or spherical pollenkit can be found on the pollen surface. It is never angular and never disappears but its amount increases during the tapetum degradation process (Pacini and Hesse 2005; Zetter and Hesse 1996). In turn, tapetum-borne lipid-origin materials with a distinct shape are represented by, e.g. viscin threads, which are stable and help in combining pollen into larger complexes in order to enhance the pollination efficiency (Rose and Barthlott 1995; Stephens et al. 2012). In contrast, the ring in *T. anomala* is ephemeral and does not persist until anther dehiscence.

In the anthesis stage in a majority of flowering plants, dehiscent anthers contain pollen grains that exhibit a varied degree of dehydration. Pollen is intensely hydrated only after deposition of grains on the stigma, and its volume increases (Wodehouse 1935). Changes in the volume of pollen grains are facilitated by the plasticity of the sporodermal wall, in particular that of the cellulose-pectin intine and the substantially thinner exine layer in the pori (Firon et al. 2012). The pollen grains in *T. anomala* are hydrated already in the anther dehiscence stage, and such pollen is transferred onto the stigma. The anatomical structure of *T. anomala* pollen promotes considerable changes in its volume, as the pollen grains have three very big pori. A single porus is composed of a thick intine layer, whereas its exine forms different-sized sporopollenin “islets” instead of a continuous layer. This sporoderm structure not only allows rapid pollen hydration but also ensures immediate activation of metabolism (Perez-Munoz et al. 1993). Our *in planta* investigations of *T. anomala* have shown that the pollen germinates in the pollen tube within several minutes after pollination. This rate of changes occurring in the initial progamic phase in the analysed species is probably associated with the exceptionally short anthesis stage, i.e. the flowers of this plant are open for 8 h and wilt afterwards. Therefore, the biology of flowering in this species is programmed in a way ensuring deposition of already hydrated pollen grains on the stigma, which largely contributes to the reproductive success. Our observations of *T. anomala* reproduction indicate that the species is capable of both vegetative and generative propagation. Sexual propagation may rely on autogamy and allogamy. The biology of the species indicates its great adaptability to changing environmental conditions.

There is a tight synchronisation of development between the tapetum and male meiotic cells. Maturation of male gametophytes is accompanied by gradual degradation of the tapetum. Products of the decomposition of this tissue are utilised for formation of a post-meiotic wall around pollen grains. The cytological image observed in this stage shows vacuolated periplasmodium, shrunken nuclei and complete degradation of organelles (Pacini and Hesse 2005). Analyses of the chemical nature of the ring aggregating *T. anomala* pollen grains imply its lipid character. Therefore, it can be assumed that it originated from flattened tapetal nuclei. This thesis is confirmed by the measurements of the sizes of the ring segments and nuclei in the periplasmodium. The sizes of the tapetal nuclei in *T. anomala* varied and ranged from 4.5 to 7.5 μm, but there were also substantially larger nuclei, which probably emerged through polyploidisation, and their sizes corresponded to the multiplied sizes of the diploid nuclei. This may explain the fact that the ring-forming segments had different sizes. In the tapetum cells, endoreplication and merging of the nuclei take place frequently. Consequently, the amount of DNA increases to 4n, 8n and even 16n, e.g. in *Cucurbita pepo* (Ciampolini et al. 1993).

To the best of our knowledge, this specific structure observed during the pollen formation process in *T. anomala* has not been described yet. In the family Comellinaceae, no pollen-aggregating structures have been described until now. The regularity of the shape and the short persistence of all the rings are puzzling. Additionally, the formation of the rings was site-specific—the rings were present both in the porus areas and in the sporodermal wall. The authors, therefore, propose the following hypotheses on the role of the ring:

1. Shock absorber hypothesis—the anther locule comprises dehydrated microspores, which increase their volume upon hydration. Given the limited space in the pollen sac, organelles, including tapetal nuclei accumulated in the plasmoid tapetum, provide mechanical protection against crushing. Due to changes in the pressure inside the anther locule, short-term merging of the nuclei and formation of the ring take place.
2. Separator hypothesis—the presence of the ring ensures isolation of individual pollen grains, thereby protecting them from formation of large aggregates. Isolation of pollen grains at the time of release thereof from the anther increases the chance of pollen deposition onto the greatest number of stigmas in separate flowers, thereby maximising their chance as a potential sexual partner.
3. Physical hypothesis—the ring is composed of segments with a lipid, hence hydrophobic, nature; the sporodermal wall contains hydrophobic structural elements as well. The lipid sources in the tapetum include plastids and endoplasmic reticulum. Possibly, the hydrophobic pollen sporoderm attracts the hydrophobic plastids or nucleus envelopes, which are arranged in a way in which the smallest area of these elements is exposed to water in the periplasmodium. The growing pressure in the locule leads to formation of large plastid aggregates, their flattening and finally arrangement in the form of a segmented ring.
